# An overview of loco-regional treatments in patients and mouse models for hepatocellular carcinoma

**DOI:** 10.1186/s13027-015-0004-2

**Published:** 2015-03-09

**Authors:** Sabrina Bimonte, Antonio Barbieri, Raffaele Palaia, Maddalena Leongito, Vittorio Albino, Mauro Piccirillo, Claudio Arra, Francesco Izzo

**Affiliations:** Hepatobiliary Unit, Istituto Nazionale per lo studio e la cura dei Tumori “Fondazione G. Pascale”, IRCCS- Via Mariano Semmola, 80131 Naples, Italy; INT Facility, Istituto Nazionale per lo studio e la cura dei Tumori “Fondazione G. Pascale”, IRCCS- Via Mariano Semmola, 80131 Naples, Italy

**Keywords:** Loco-regional treatments, Hepatocellular carcinoma, Drug delivery, Safety, Efficacy

## Abstract

Hepatocellular carcinoma is a highly aggressive malignancy and is the third leading cause of cancer-related deaths worldwide. Although surgery is currently considered the most effective curative treatment for this type of cancer, it is note that most of patients have a poor prognosis due to chemioresistence and tumor recurrence. Loco-regional therapies, including radiofrequency ablation, surgical resection and transcatheter arterial chemoembolization play a major role in the clinical management of hepatocellular carcinoma. In order to improve the treatment outcome of patients diagnosed with this disease, several in *vivo* studies by using different techniques on cancer mouse models have been performed. This review will focus on the latest papers on the efficacy of loco-regional therapy and combined treatments in patients and mouse models of hepatocellular carcinoma.

## Introduction

Hepatocellular carcinoma (HCC) is a worldwide malignancy and the third leading causes of cancer-related deaths [1,2]. The incidence of primary liver cancer is increasing in several developed countries and the increase will likely continue for some decades as a result of viral infection of hepatitis C [3,4]. Liver transplant and surgery are considered the potential effective curative treatment for HCC, although most patients have a poor prognosis due to unresectable disease at presentation, multidrug resistance (MDR) [5] and tumor recurrence. In most cases, this pathology develops in patients with chronic liver disease (70-90% of all patients) [6]. In order to bypass these problems, several regional cancer therapy and multimodality treatments, have been developed [7,8]. Additionally, to improve the treatment outcome of patients diagnosed with HCC, several in *vivo* studies by using different techniques on HCC mouse models have been performed. This review will focus on the latest papers on the efficacy of loco-regional therapy and combined treatments in patients and mouse models of hepatocellular carcinoma.

### Loco-regional treatments in patients with hepatocellular carcinoma

Loco-regional therapies, including image-guided tumor ablation, percutaneous ethanol injection (PEI), transcatheterial chemoembolization (TACE) and transarterial radioembolization (TARE) are commonly used as a nonsurgical approach for HCC patients [9-11]. For patients with early-stage unresectable HCC, image-guided tumor ablation (chemical or thermal) is recommended. Chemical ablation is used for treatment of nodular-type HCC and it is based on PEI which leads to tumor necrosis. One limit of PEI is represented by tumor recurrence in HCC patients as well as needs of multiple sessions [12,13]. Acetic acid injection is considered an alternative to PEI for chemical ablation of HCC, although it is not commonly used due to lower survival outcomes of patients [14]. Among thermal ablative therapies used in clinical practice, radiofrequency ablation (RFA) which induces thermal injury to the cancer tissue through electromagnetic energy deposition, is considered as the standard treatment for local ablation of HCC due to its anticancer effects and survival benefit for patients [6,15-22]; . On the contrary, several clinical studies have demonstrated that radiofrequency ablation for HCC increased risk of local tumor progression and incomplete ablation [23-26]. To bypass these problems, novel thermal techniques (microwave ablation; MWA, laser ablation and cryoablation) [27-29] and non-thermal techniques (reversible electroporation ECT, irreversible electroporation IRE and light-activated drug therapy), for HCC tumor ablation have been developed. Clinical studies show that non-thermal techniques seem to overcome the limitations of chemical and thermal-based techniques in the treatment of HCC [30,31]. Another approach used to noninvasive multinodular HCC tumors at the intermediate stage, is TACE, which belongs to image-guided transcatheter tumor therapy. This technique is based on an intra-arterial infusion of a drug (mainly cisplatin or doxorubicin) with or without a viscous emulsion, followed by embolization of the blood vessel with embolic agents that leads to ischemia and cytotoxic effects or liver internal radiation using yttrium-90 (^90^Y) spheres. There are two types of TACE; the first one is called conventional TACE that consists in the administration of an anticancer in lipiodol emulsion followed by embolic agents [16,24,25,32]; and the second one, is called TACE with drug-eluting beads that uses embolic microspheres that release the drug in the sustained-released system [33,34]. Several studies have demonstrated that TACE with drug-eluting beads significantly increases efficacy and safety for patients respect to conventional TACE [35,36]. A new technique that can be considered a potential treatment for patients with HCC alternative to TACE, is TARE. This approach consists in the infusion of radioactive substances including microsphere containing yittrium-90 (^90^Y) or similar agents, into hepatic artery [37-39]. By using this technique, these microspheres will be delivered to the area which surrounds the tumor, with low-penetration to the tumor itself. Several clinical studies have demonstrated that radioembolization treatment with ^90^Y can be safely used in patients with HCC [40,41], although this technique leads to several possible side-effects (gastric ulceration, pancreatitis, radiation pneumonitis, etc.). Further investigations will be necessary in the setting of randomized controlled trials (RCT). It is important to underline that any loco-regional treatment described above, summarized in Table 1, leads to a high rate of tumor recurrence in patients. For this reason, new combined treatments for HCC have been developed. These combined strategies are focused on the synergy between molecular targeted drugs (i.e. sorafenib, etc.) and loco-regional treatments [42-46]. Clinical trials on these new techniques are currently ongoing and can be used as therapy of election for patients with HCC.

**Table 1:** **Effects of Loco-regional treatment on patients with hepatocellular carcinoma**

**Treatment**	**Effects on tumor**	**Side-effects on HCC patients**	**Reference**
**PEI**	*Tumor necrosis*	*High local recurrence rate*	[12,13]
**Acetic acid acetic injection**	*Glypican-3, RFA*	*Lower survival outcome of patients*	[14]
**RFA**	*Thermal injury to the cancer tissue*	*Increased risk of local tumor progression and incomplete ablation*	[6,15-19,23-26,47-50]
**MWA**	*Tumor destruction*	*High local recurrence rate*	[27]
**Cryoablation**	*Tumor necrosis*	*Severe coagulopathy*	[29]
**Laser Ablation**	*Tumor necrosis*	*Lower survival outcome of patients*	[28]
**IRE**	*Cancer tissue death*	*High local recurrence rate*	[30,31]
**Yttrium-90 Radioembolization**	*Tumor necrosis Cancer tissue death Apoptotic cell death and permanent tumor blood vessel closure*	*Transient liver insufficiency, ascitesHigh local recurrence rate High local recurrence rate*	[30,40,41,51]
**ECT Light-activated drug therapy**			
**TACE**	*Tumor necrosis*	*High rate of tumor recurrence in patients*	[16,24,25,32-34,37-39]

### The efficacy of Loco-regional treatment in mouse models of hepatocellular carcinoma

Loco-regional therapies are considered the best treatments in patients with unresectable HCC. One of the principal obstacles implicated in their unsuccessful therapy is MDR. In order to improve the treatment outcome of patients diagnosed with HCC, several in *vivo* studies by using loco-regional techniques and combined treatments on HCC mouse models have been performed. The first study that tested an effective strategy for the treatment of HCC with MDR, demonstrated that chemicals in combination with adriamycin (ADM), mitomycin, 5-fluoruracil (5-FU), mutant human tumor necrosis factor-α (rmhTNF-α) and hydroxyapatite nanoparticles (nHAPs), could be beneficial for the local treatment of advanced HCC in vitro and *in vivo* experimental conditions. Specifically, it has been showed that the chemicals acted in synergism with rmhTNF-α and nHAP in suppressing the growth of human hepatoma MDR liver hepatocellular (HepG2)/ADM cells by inducing apoptosis and by reducing tumor growth in liver hepatocellular mouse model [52]. Another group demonstrated that Glypican-3 (GPC3), a carcinoembryonic antigen, could be considered as an ideal target for anticancer immunotherapy against HCC. In this study, the authors compared the induction of the GPC3-specific T-cell-mediated immune response after loco regional therapies, such as RFA or TACE in HCC patients and tumor-bearing mice [53,54]. Recently has been developed a new bioelectrical technology in cancer therapy, the nanosecond pulsed electric field (nsPEF). NsPEF can generate pulsed high voltage electric field in ultra-short nanosecond duration, to produce immediate power which could ablate targeted tumor [54]. It has been reported that nsPEF treatment, is efficient to control hepatocellular carcinoma growth in HCC mouse model. In this study, was investigated the use of nsPEF on a human HCC cell lines and a high pulmonary metastatic potential HCC xenograft mouse model (HCCLM3). The multiple fractionated dose of nsPEFs efficiently inhibited tumors without increasing the risk of secondary metastasis, indicating that nsPEF can be used as a loco-regional therapy for hepatocellular carcinoma [55]. Recently it has been demonstrated that targeted gold nanoconjugates in combination with RF halted the growth of subcutaneous human hepatoma (Hep3B) xenografts. These xenografts also demonstrated increased apoptosis, necrosis and decreased proliferation compared to controls [56]. Taken together all these different data, summarized in Table 2, suggest that these combined treatments could represent new methods to deliver effective and safe therapies to patients with advanced HCC.

**Table 2:** **Effects of Loco-regional treatment on tumor growth in mouse models of hepatocellular carcinoma**

**Study model**	**Treatment**	**Effects on tumor**	**Reference**
HepG2/ADM human hepatoma mouse model	***nHAPs, rmhTNF-alpha, ADM, mitomycin, 5-FU***	***Tumor growth reduction***	[52]
GPC3-negative colorectal adenocarcinoma mouse model	***Glypican-3, RFA***	***Tumor growth reduction***	[53,54]
HCCLM3 mouse model	***nsPEF***	***Tumor growth reduction***	
Hep3B xenograft mouse model	***5 Gold nanoparticles, RF***	***Tumor growth reduction***	[56]

## Abbreviations

HCC: Hepatocellular carcinoma
MDR: Multidrug resistance
PEI: Percutaneous ethanol injection
TACE: Transcatheter arterial chemoembolization
TARE: Radioembolization
RFA: Radiofrequency ablation
MWA: Microwave ablation
ECT: Reversible electroporation
IRE: Irreversible electroporation
^90^Y: Yittrium-90
RCT: Randomized controlled trials
ADM: Adriamycin
5-FU: 5-Fluoruracil
rmhTNF-alpha: Recombinant mutant human tumour necrosis factor-alpha
nHAP: Hydroxyapatite nanoparticles
HepG2: Liver hepatocellular carcinoma
GPC-3: Glypican-3
nsPEF: Nanosecond pulsed electric field
RF: Radiofrequency
HCCLM3: High metastatic hepatocellular carcinoma
Hep3b: Human hepatoma 3b
